# Supplementary material for: Adherence and Dietary Composition during Intermittent vs. Continuous Calorie Restriction: Follow-Up Data from a Randomized Controlled Trial in Adults with Overweight or Obesity
Source: Nutrients. 2021 Apr 5;13(4):1195. doi: 10.3390/nu13041195 (PMC8067073; doi:10.3390/nu13041195)
Supplement: Supplementary file 1 [file nutrients-13-01195-s001.pdf]

Adherence and Dietary Composition During Intermittent vs. Continuous Calorie Restriction: Follow-Up Data from a Randomized Controlled Trial in Adults with Overweight or Obesity

Supplementary Methods

Calculation of the average intakes in the ICR and the CCR group

For the CCR group (at all four time points: wk0, wk2, wk12, wk50) and for the ICR group at wk0, the average mean intake per day was automatically calculated by PRODI 6.8, by dividing the sum of the total intake of the week by seven. At wk2, wk12, and wk50 protocols of the ICR group were divided into two separate protocols, with one covering the intake on NR days and the other including R days only. In order to assess the weekly average of the daily intakes, the sum of the average intake on NR days was multiplied by five and on R days by two, and then divided by seven. A brief overview of the underlying calculation for the average energy, nutrient and food intakes is given below (Table S1).

Table S1. Formula for the calculation of the average energy, nutrient and food intake per day and on non-restricted and restricted days. [1]

Average		Calculation
Daily[2]	=	$\frac{\sum \text{Intake on 7 days}}{7}$
NR days[3]	=	$\frac{\sum \text{Intake on 5 NR days}}{5}$
R days[3]	=	$\frac{\sum \text{Intake on 2 R days}}{2}$
Daily (ICR)[3]	=	$\frac{\text{ØNR days} * 5 + \text{ØR days} * 2}{7}$

[1]CCR, continuous calorie restriction; ICR, intermittent calorie restriction; NR, non-restricted; R, restricted.
[2]CCR at wk0, wk2, wk12 and wk50; ICR at wk0.
[3]ICR group only (at wk2, wk12 and wk50) weighed by the reported number of days that each participant conducted R days.

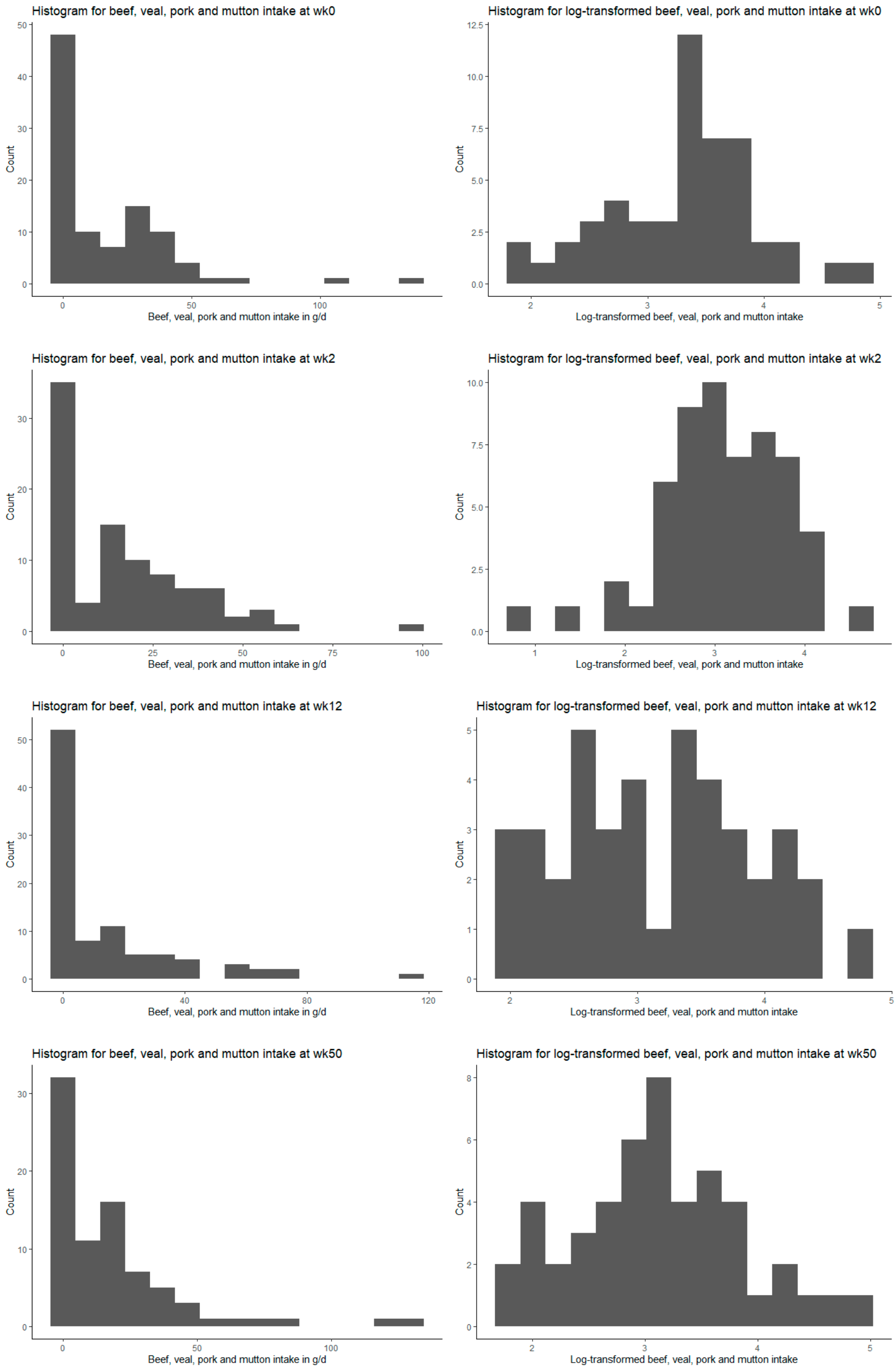

Figure S1. Data-distribution of the crude and the log-transformed intake data at wk0, wk2, wk12 and wk50 by the example of the food group “beef, veal, pork and mutton”. P-values calculated by the Shapiro-Wilk test were significant for the crude intake data (wk0, wk2, wk12 and wk50: $p < 0.05$) and non-significant for the log-transformed intake data (wk0: $p = 0.30$, wk2: $p = 0.061$, wk12: $p = 0.34$, wk50: $p = 0.46$) at all timepoints.

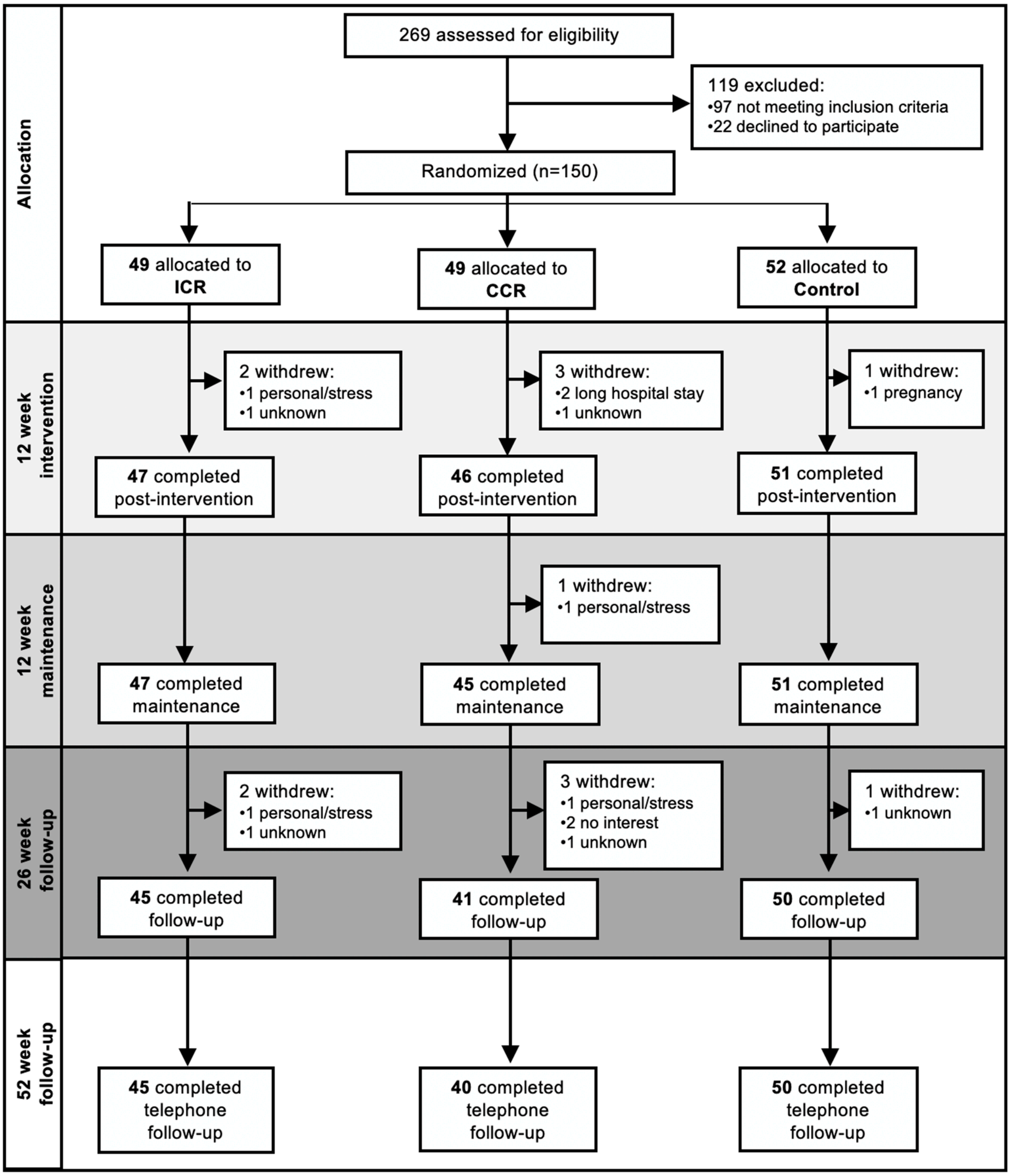

Figure S2. CONSORT diagram on the HELENA Trial from baseline to wk102 adapted from Schübel et al. 2018 (1).

Supplemental Tables

Table S2. Percentage of reported energy intakes at wk2, wk12 and wk50 in relation to the reported baseline energy intake and to the calculated isoenergetic energy requirement. [1]

		wk2	P-value[2]	wk12	P-value[2]	wk50	P-value[2]
Energy [% from reported baseline energy intake]							
**CCR**	Daily	79.6 ± 18.8	0.98	78.6 ± 15.0	0.94	91.7 ± 38.9	0.88
**ICR**	Daily	79.4 ± 36.6		78.1 ± 38.6		90.4 ± 36.3	
	NR days	94.4 ± 39.0		92.2 ± 47.8		94.3 ± 37.6	
	R days	42.0 ± 38.2		40.4 ± 27.4		35.6 ± 28.6	
Energy [% from calculated isoenergetic energy requirement[3]]							
**CCR**	Daily	61.2 ± 11.3	0.07	61.3 ± 13.1	0.07	70.2 ± 17.6	0.26
**ICR**	Daily	56.0 ± 15.6		55.3 ± 17.6		65.1 ± 21.4	
	NR days	67.8 ± 21.8		65.3 ± 20.2		67.9 ± 21.4	
	R days	26.9 ± 8.4		26.9 ± 10.4		21.6 ± 7.6	

[1]Values are means ± SDs [%] unless otherwise indicated. Data were included from 98 participants (wk2 n = 45 for ICR, n = 46 for CCR; wk12 n = 47 for ICR [R days: n = 44], n = 46 for CCR; wk50 n = 41 for ICR [R days: n = 12], n = 38 for CCR). CCR, continuous calorie restriction; ICR, intermittent calorie restriction; NR, non-restricted; R, restricted.
[2]P-values for differences in daily energy intake between groups were calculated by ANOVA F-test.
[3]Isoenergetic energy requirement was calculated by the Harris-Benedict equation multiplied with the PAL.

Table S3. Measured weight loss vs. predicted weight loss according to the reported energy deficit during the intervention phase. [1]

	Weight [kg] wk0	Weight [kg] wk12	Measured weight loss [kg]	Predicted weight loss [kg][2]
**ICR**	96.4 ± 15.8	90.2 ± 15.9	-6.5 ± 4.8	-8.2 ± 3.9
**CCR**	92.5 ± 15.7	87.3 ± 14.3	-4.7 ± 3.5	-6.7 ± 2.3

[1]Values are means ± SDs unless otherwise indicated. Changes are compared to baseline values and are shown as absolute differences [kg]. wk0 n = 49 for ICR and CCR; wk12 n = 47 for ICR, n = 46 for CCR. CCR, continuous calorie restriction; ICR, intermittent calorie restriction.
[2]Weight loss is calculated based on the energy deficit in relation to the isoenergetic energy requirement (2).

Table S4. Estimated isoenergetic energy requirement and reported energy intakes at wk0, wk12 and wk50. [1]

	Energy Requirement wk0 [kcal/d][2]	Reported intake wk0 [kcal/d]	Energy Requirement wk12 [kcal/d] [2]	Reported intake wk12 [kcal/d]	Energy Requirement wk50 [kcal/d] [2]	Reported intake wk50 [kcal/d]
**ICR**	2630.8 ± 490.9	2053.3 ± 746.0	2528.7 ± 457.0	1438.4 ± 486.1	2561.6 ± 489.9	1689.8 ± 624.4
**CCR**	2507.3 ± 378.5	1981.0 ± 476.5	2435.4 ± 355.7	1529.4 ± 364.8	2447.8 ± 353.8	1768.8 ± 553.2

[1]Values are means ± SDs unless otherwise indicated. Data were included from 98 participants (wk0 n = 49 for ICR and CCR; wk12 n = 47 for ICR, n = 46 for CCR; wk50 n = 41 for ICR, n = 38 for CCR). CCR, continuous calorie restriction; ICR, intermittent calorie restriction.
[2]Isoenergetic energy requirement was calculated by the Harris-Benedict equation multiplied with the PAL (Baseline body height was used for the calculation of all timepoints).

Table S5. Changes in body weight by intervention group between baseline (wk0) and two years after baseline (wk102). [1]

	Weight [kg] wk0	Weight [kg] wk12	Change [%][1] wk0-12	Weight [kg] wk24	Change [%][1] wk0-24	Weight [kg] wk50	Change [%][1] wk0-50	Weight [kg] wk102	Change [%][1] wk0-102
**ICR**	96.4 ± 15.8	90.2 ± 15.9	-7.1 ± 0.7*	90.1 ± 16.4	-7.1 ± 0.9*	92.0 ± 17.1	-5.2 ± 1.2*	92.7 ± 16.7	-4.3 ± 1.0*
**CCR**	92.5 ± 15.7	87.3 ± 14.3	-5.3 ± 0.6*	87.3 ± 14.0	-5.6 ± 0.9*	87.9 ± 14.4	-4.9 ± 1.1*	87.3 ± 14.5	-5.0 ± 1.1*
P-value ICR vs. CCR [2]			0.053		0.29		0.92		0.63

[1]Values are means ± SDs unless otherwise indicated. Changes are compared to baseline values and are shown as means of $\log_e$ percentage changes ± standard error of log percentage changes (%).
*Significant within group change over time compared to baseline at $p < 0.05$ from paired t-test. Data were included from 98 participants (wk0 n = 49 for ICR and CCR; wk12 n = 47 for ICR, n = 46 for CCR; wk24 n = 46 for ICR, n = 41 for CCR; wk50 n = 45 for ICR, n = 41 for CCR; wk50 n = 45 for ICR, n = 39 for CCR). CCR, continuous calorie restriction; ICR, intermittent calorie restriction.
[2]P-values for time-treatment interactions were calculated with linear mixed models adjusted for age and sex.

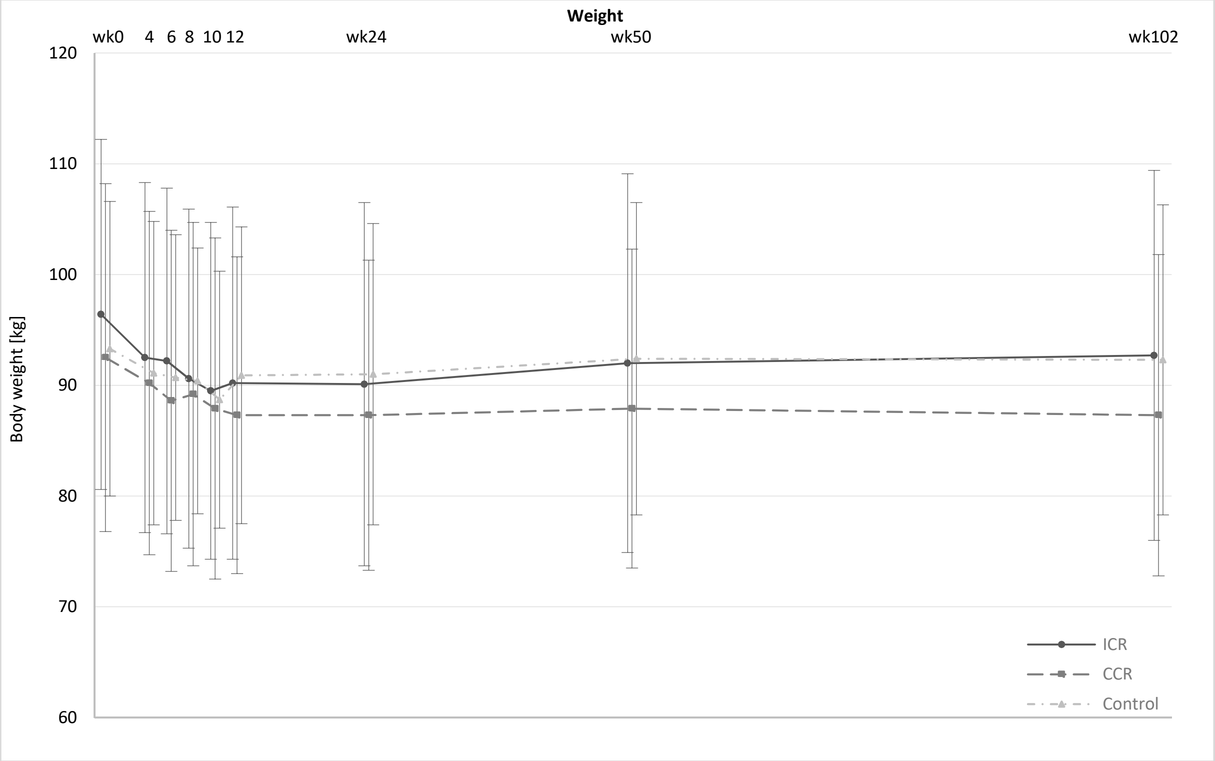

Figure S3. Self-reported (wks 4, 6, 8, 10, 102) and measured (wks 0, 12, 24, 50) body weight by study group from baseline to wk102. Data are shown as means ± SDs (n = 49 at baseline in both intervention groups; n = 52 in the control group). Results on anthropometric parameters including body weight at baseline (wk0) and wk12, wk24 and wk50 have been published in detail before (1). Body weight from these time points is shown here again to facilitate a better interpretation of body weight at the 2-y follow up assessment (wk102). CCR, continuous calorie restriction; ICR, intermittent calorie restriction.

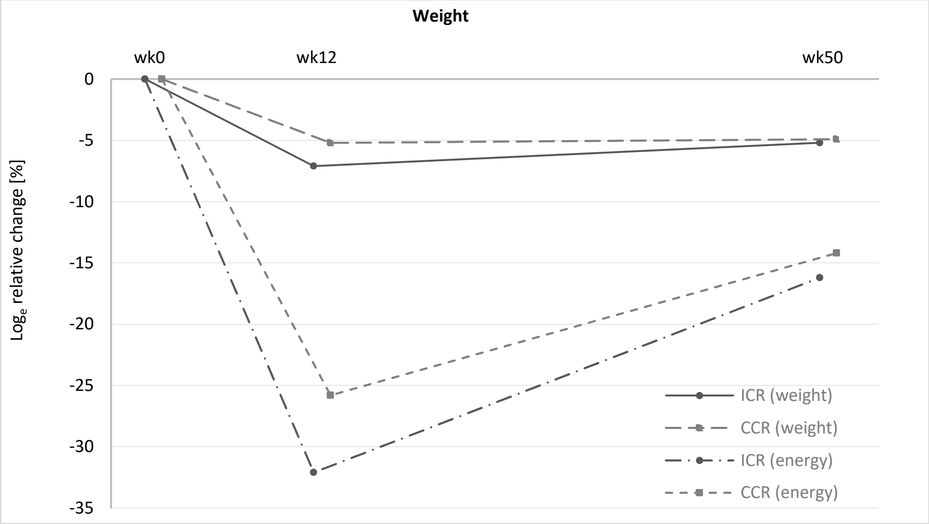

Figure S4. Relative changes in body weight and energy intake by intervention group from baseline to wk12 and wk50. Data are shown as means of $\log_e$ percentage changes, with baseline values as the reference for the ICR (n = 49) and the CCR group (n = 49). CCR, continuous calorie restriction; ICR, intermittent calorie restriction.

Table S6. Questionnaire data on self-reported dietary behavior during the year after the end of the study. [1]

	ICR	CCR	P-value[2]
Have you tried to continue with the diet assigned to you in last year’s study?			
yes, mostly	4 (8.9)	15 (37.5)	<.001
yes, more often	9 (20.0)	12 (30.0)	
yes, rarely	19 (42.2)	11 (27.5)	
no	13 (28.9)	2 (5.0)	
What type(s) of diet have you continued with?			
- CCR			
yes	5 (11.1)	38 (95.0)	<.001
no	40 (88.9)	2 (5.0)	
- “Healthy balanced diet” (according to the DGE recommendations)			
yes	21 (46.7)	18 (45.0)	0.88
no	24 (53.3)	22 (55.0)	
- “5:2 diet” (ICR)			
yes	32 (71.1)	1 (2.5)	<.001
no	13 (28.9)	39 (97.5)	
- “6:1 diet” (ICR)			
yes	0 (0.0)	0 (0.0)	.
no	45 (100.0)	40 (100.0)	
- Resumed old behavior patterns (without watching one's weight)			
yes	36 (80.0)	33 (82.5)	0.77
no	9 (20.0)	7 (17.5)	

[1]Values are counts (% of answers) unless otherwise indicated. Data were included from 85 participants (ICR n = 45; CCR n = 40). CCR, continuous calorie restriction; DGE, Deutsche Gesellschaft für Ernährung (German Nutrition Society); ICR, intermittent calorie restriction.
[2]P-values were calculated by Fisher's exact test (2-Tail).

Table S7. Weight change according to self-reported continuation of the intermittent calorie restriction and the continuous calorie restriction diet. [1]

	wk0	wk12	Change [%][1] wk0-wk12	wk24	Change [%][1] wk0-wk24	wk50	Change [%][1] wk0-wk50	wk102	Change [%][1] wk0-wk102
Weight [kg]									
**ICR continuation**									
yes	98.1 ± 16.0	91.6 ± 15.6	-6.9 ± 0.9*	91.5 ± 15.8	-7.1 ± 1.1*	92.5 ± 16.7	-6.1 ± 1.5*	92.9 ± 16.7	-5.7 ± 1.2*
no	93.0 ± 17.2	86.3 ± 17.7	-7.8 ± 1.7*	87.2 ± 18.7	-7.0 ± 1.8*	90.6 ± 18.7	-3.0 ± 1.9	92.3 ± 17.5	-1.0 ± 1.5
P-value[2]			0.86		0.71		0.19		0.025
**CCR continuation**									
yes	92.1 ± 14.7	87.3 ± 13.5	-5.2 ± 0.6*	87.2 ± 13.5	-5.3 ± 0.9*	87.6 ± 13.9	-4.9 ± 1.2*	87.7 ± 14.6	-5.0 ± 1.2*
no	85.6 ± 19.5	78.1 ± 21.7	-9.9 ± 3.7	77.5 ± 20.5	-10.4 ± 2.7	80.9 ± 20.1	-5.9 ± 1.5	81.0 ± 12.7	-4.8 ± 5.1
P-value[2]			-		-		-		-

[1]Values are means ± SDs unless otherwise indicated. Changes are compared to baseline values and are shown as means of $\log_e$ percentage changes ± standard error of log percentage changes (%).
*Significant within group change over time compared to baseline at $p < 0.05$ from paired t-test. ICR-yes n = 32, ICR-no n = 13; CCR-yes n = 38, CCR-no n = 2. CCR, continuous calorie restriction; ICR, intermittent calorie restriction.
[2]P-values for time-treatment interactions (yes vs. no) were calculated with linear mixed models adjusted for age and sex.
Test not used for CCR given that only n = 2 reported to not have continued with CCR.

Table S8. Dietary habits during the year after the end of the study other than intermittent calorie restriction ("5:2 diet") and continuous calorie restriction. [1]

	ICR	CCR
Followed a diet other than ICR ("5:2 diet") or CCR during the year after the end of the study		
yes	11 (24.4)	4 (10.0)
no	34 (75.6)	36 (90.0)
What type(s) of diet did you follow? [2] (one bullet point per participant)	**ICR "non-continuers"**	**CCR "non-continuers"**
	• No eating after 6 pm (9wks)<br>• Abstain from industrially produced sweets (10wks)<br>• Low carb diet in the evening (10wks)	-
	**ICR "continuers"**	**CCR "continuers"**
	• "16:8" ICR diet (26wks)<br>• "Metabolic diet" (2wks)<br>• Weight Watchers (24wks)<br>• Protein-rich diet; Protein powder / diet shakes (4wks)<br>• Protein-rich low carb diet (17wks)<br>• Low carb diet (3wks)<br>• Low carb diet (10wks)<br>• Low carb diet (20wks)	• "Alkali fasting" (3wks)<br>• Weight Watchers (4wks)<br>• Low carb diet in the evening (12wks)<br>• Low carb diet (2wks)

[1]Values are counts (% of answers) unless otherwise indicated. Data were included from 85 participants (ICR n = 45; CCR n = 40). CCR, continuous calorie restriction; ICR, intermittent calorie restriction.
[2]Type of diet followed by participants who reported following a diet other than ICR or CCR in the year after the end of the study, divided into those who reported also continuing the diet assigned to them in the study during the year vs. not continuing. Duration of the diet was reported by participants in number of wks out of the 52wks.

Table S9. Weight change according to self-reported continuation of a "healthy balanced diet" during intermittent calorie restriction and continuous calorie restriction. [1]

	wk0	wk12	Change [%][1] wk0-wk12	wk24	Change [%][1] wk0-wk24	wk50	Change [%][1] wk0-wk50	wk102	Change [%][1] wk0-wk102
Weight [kg]									
**ICR** – Continuation of the "healthy balanced diet"									
yes	93.3 ± 14.6	85.5 ± 12.5	-8.6 ± 1.1*	84.5 ± 11.7	-9.6 ± 1.4*	86.9 ± 13.0	-7.1 ± 1.9*	87.8 ± 13.6	-6.1 ± 1.6*
no	99.5 ± 17.5	94.2 ± 18.1	-5.9 ± 1.0*	95.2 ± 18.7	-4.8 ± 1.1*	96.4 ± 19.2	-3.6 ± 1.4*	97.0 ± 18.2	-2.8 ± 1.2*
P-value[2]			0.10		0.01		0.12		0.10
**CCR** – Continuation of the "healthy balanced diet"									
yes	93.9 ± 16.8	89.3 ± 15.6	-5.0 ± 1.0*	88.9 ± 16.0	-5.4 ± 1.6*	88.9 ± 16.0	-5.5 ± 2.2*	88.8 ± 16.0	-5.6 ± 1.8*
no	90.0 ± 12.9	84.9 ± 12.0	-5.8 ± 0.8*	84.9 ± 11.7	-5.7 ± 1.0*	86.0 ± 12.4	-4.5 ± 1.0*	86.1 ± 13.3	-4.4 ± 1.5*
P-value[2]			0.72		0.99		0.60		0.51

[1]Values are means ± SDs unless otherwise indicated. Changes are compared to baseline values and are shown as means of log_e percentage changes ± standard error of log percentage changes (%).
*Significant within group change over time compared to baseline at $p < 0.05$ from paired t-test. ICR-yes n = 21, ICR-no n = 24; CCR-yes n = 18, CCR-no n = 22. CCR, continuous calorie restriction; ICR, intermittent calorie restriction.
[2]P-values for time-treatment interactions (yes vs. no) were calculated with linear mixed models adjusted for age and sex.

Table S10. Changes in macronutrient intake [g/d] by intervention group. [1]

		wk0	wk2	Change [%][1] wk0-wk2	P-value[2]	wk12	Change [%][1] wk0-wk12	P-value[2]	wk50	Change [%][1] wk0-wk50	P-value[2]
Protein [g/d]	**ICR**	76.6 ± 27.9	68.1 ± 19.9	-7.5 ± 5.6	0.99	63.2 ± 24.3	-17.1 ± 5.3*	0.76	67.2 ± 27.7	-8.3 ± 5.0	0.89
	**CCR**	72.7 ± 20.4	65.0 ± 16.2	-12.0 ± 4.7*		62.1 ± 13.3	-16.1 ± 4.0*		67.6 ± 19.3	-10.0 ± 5.7	
	P-value[3]	0.44	0.41			0.79			0.95		
Fat [g/d]	**ICR**	86.0 ± 37.9	48.6 ± 18.2	-52.5 ± 6.9*	0.24	50.4 ± 22.5	-50.7 ± 6.2*	0.19	63.7 ± 27.4	-24.9 ± 7.5*	0.71
	**CCR**	79.5 ± 24.4	49.8 ± 15.6	-46.9 ± 5.6*		52.4 ± 16.8	-41.7 ± 5.1*		62.7 ± 31.4	-26.2 ± 8.0*	
	P-value[3]	0.32	0.73			0.64			0.88		
Carbohydrates [g/d]	**ICR**	215.3 ± 80.7	165.5 ± 56.1	-23.9 ± 5.5*	0.08	157.7 ± 50.9	-27.2 ± 5.8*	0.06	186.9 ± 74.7	-13.1 ± 5.7*	0.34
	**CCR**	211.7 ± 54.1	183.0 ± 42.4	-14.3 ± 3.4*		178.1 ± 49.5	-17.5 ± 3.5*		205.5 ± 82.2	-8.1 ± 6.0	
	P-value[3]	0.80	0.10			0.05			0.30		
Total sugar [g/d]	**ICR**	82.7 ± 43.1	67.1 ± 29.9	-19.2 ± 6.6*	0.42	63.9 ± 24.6	-21.7 ± 6.1*	0.71	70.1 ± 32.4	-17.5 ± 6.6*	0.26
	**CCR**	84.5 ± 30.8	74.9 ± 22.6	-9.1 ± 6.0		68.4 ± 24.3	-18.6 ± 6.2*		88.2 ± 67.9	-2.3 ± 9.1	
	P-value[3]	0.82	0.16			0.38			0.13		
Fiber [g/d] [4]	**ICR**	17.9 ± 8.1	21.8 ± 7.4	25.1 ± 6.4*	0.07	18.9 ± 7.9	8.4 ± 7.2	0.08	19.8 ± 8.9	12.0 ± 7.4	0.31
	**CCR**	17.5 ± 6.1	24.4 ± 7.7	33.6 ± 4.9*		21.8 ± 9.1	22.3 ± 5.0*		21.9 ± 12.4	19.3 ± 6.9*	
	P-value[3]	0.79	0.11			0.11			0.39		
Fiber [E%]	**ICR**	1.8 ± 0.6	3.2 ± 0.8	62.6 ± 5.4*	0.40	2.9 ± 0.9	49.9 ± 5.4*	0.66	2.5 ± 0.8	31.2 ± 6.4*	0.98
	**CCR**	1.8 ± 0.6	3.2 ± 0.8	57.3 ± 4.5*		2.9 ± 1.0	46.9 ± 4.7*		2.5 ± 0.9	32.6 ± 6.6*	
	P-value[3]	0.73	0.60			0.79			0.99		
Alcohol [g/d]	**ICR**	9.1 ± 10.7	5.5 ± 10.3	-119.5 ± 29.6*	0.12	6.9 ± 9.6	-57.7 ± 25.8*	0.06	6.7 ± 7.8	-69.0 ± 32.3*	0.41
	**CCR**	11.0 ± 12.9	4.5 ± 6.8	-133.7 ± 39.4*		5.3 ± 7.6	-122.9 ± 36.4*		7.3 ± 9.6	-68.9 ± 30.2*	
	P-value[3]	0.42	0.61			0.39			0.78		

[1]Values are means ± SDs unless otherwise indicated. Changes are compared to baseline values and are shown as means of $\log_e$ percentage changes ± standard error of log percentage changes (%). *Significant within group change over time compared to baseline at $p < 0.05$ from paired t-test. Data were included from 98 participants (wk0 n = 49 for ICR and CCR; wk2 n = 45 for ICR, n = 46 for CCR; wk12 n = 47 for ICR, n = 46 for CCR; wk50 n = 41 for ICR, n = 38 for CCR). CCR, continuous calorie restriction; ICR, intermittent calorie restriction.

[2]P-values for time-treatment interactions were calculated with linear mixed models adjusted for age and sex.

[3]P-values for differences between groups at each time point were calculated by ANOVA F-test.

[4]The ratio of insoluble to soluble fiber was approximately 2:1 in both groups and was constant over time.

Table S11. Changes in micronutrient intake by intervention group. [1]

	wk0	wk2	Change [%][1] wk0-wk2	P-value[2]	wk12	Change [%][1] wk0-wk12	P-value[2]	wk50	Change [%][1] wk0-wk50	P-value[2]
Vitamin A (Retinol) [mg/d]										
**ICR**	0.5 ± 0.4	0.6 ± 1.4	-23.3 ± 14.5	0.78	0.5 ± 1.3	-48.6 ± 14.9*	0.40	0.4 ± 0.5	-33.9 ± 14.9*	0.90
**CCR**	0.4 ± 0.3	0.4 ± 0.8	-30.3 ± 15.2		0.2 ± 0.1	-64.9 ± 11.7*		0.3 ± 0.2	-33.2 ± 10.2*	
β-carotene [mg/d]										
**ICR**	2.8 ± 2.3	8.1 ± 5.8	105.8 ± 13.4*	0.012	6.1 ± 5.0	65.3 ± 15.6*	0.06	4.4 ± 3.8	29.7 ± 16.1	0.81
**CCR**	3.4 ± 2.2	5.9 ± 3.7	57.6 ± 13.4*		4.9 ± 3.3	37.5 ± 13.8*		4.5 ± 3.9	17.0 ± 14.2	
α-tocopherol [mg/d]										
**ICR**	8.4 ± 4.2	8.3 ± 2.7	3.4 ± 7.7	0.08	7.5 ± 3.4	-9.8 ± 8.1	0.12	7.8 ± 2.8	-5.5 ± 8.8	0.41
**CCR**	8.6 ± 2.8	9.9 ± 3.7	16.4 ± 7.1*		9.0 ± 3.2	6.7 ± 6.7		8.5 ± 3.8	2.8 ± 6.9	
Vitamin C [mg/d]										
**ICR**	95.8 ± 58.2	139.1 ± 70.2	44.3 ± 10.6*	0.37	125.6 ± 61.9	35.8 ± 11.4*	0.61	126.1 ± 75.7	28.1 ± 12.2*	0.99
**CCR**	96.6 ± 48.0	151.9 ± 66.2	46.5 ± 8.4*		134.5 ± 74.7	31.7 ± 8.2*		124.2 ± 68.7	25.6 ± 8.8*	
Vitamin D [µg/d]										
**ICR**	2.6 ± 4.2	2.6 ± 2.3	11.0 ± 15.9	0.58	1.7 ± 1.5	-33.3 ± 15.5*	0.46	1.9 ± 1.9	-8.3 ± 15.1	0.28
**CCR**	2.0 ± 1.2	1.7 ± 1.3	-27.2 ± 12.8*		1.6 ± 1.1	-27.2 ± 9.0*		2.5 ± 3.5	-10.2 ± 12.8	
Thiamin [mg/d]										
**ICR**	1.0 ± 0.4	1.1 ± 0.3	8.2 ± 6.7	0.62	0.9 ± 0.3	-5.6 ± 6.5	0.94	1.0 ± 0.4	1.5 ± 5.7	0.61
**CCR**	1.0 ± 0.5	1.1 ± 0.4	8.1 ± 5.9		0.9 ± 0.2	-6.6 ± 5.6		1.0 ± 0.4	-5.2 ± 8.1	
Riboflavin [mg/d]										
**ICR**	1.2 ± 0.5	1.3 ± 0.4	9.2 ± 4.9	0.78	1.1 ± 0.5	-8.9 ± 4.6	0.88	1.1 ± 0.5	-6.9 ± 4.8	0.31
**CCR**	1.1 ± 0.3	1.3 ± 0.3	10.1 ± 5.7		1.1 ± 0.3	-8.1 ± 5.0		1.2 ± 0.3	2.5 ± 5.8	
Niacin [mg/d]										
**ICR**	27.7 ± 10.1	28.8 ± 8.4	9.1 ± 5.6	0.85	24.4 ± 9.6	-10.9 ± 4.6*	0.99	25.5 ± 1.1	-3.6 ± 4.5	0.71
**CCR**	26.7 ± 8.3	27.9 ± 6.7	4.4 ± 4.6		24.0 ± 5.8	-10.4 ± 4.7*		27.1 ± 8.7	-2.8 ± 5.5	
Vitamin B_6 [mg/d]										
**ICR**	1.3 ± 0.5	1.5 ± 0.5	12.8 ± 6.1*	0.59	1.3 ± 0.4	-0.9 ± 6.2	0.54	1.3 ± 0.5	3.1 ± 6.6	0.78
**CCR**	1.3 ± 0.4	1.5 ± 0.5	14.0 ± 5.1*		1.3 ± 0.4	2.5 ± 5.2		1.4 ± 0.5	4.4 ± 5.8	

	wk0	wk2	Change [%][1] wk0-wk2	P-value[2]	wk12	Change [%][1] wk0-wk12	P-value[2]	wk50	Change [%][1] wk0-wk50	P-value[2]
Folate [µg/d]										
**ICR**	189.3 ± 79.7	253.9 ± 76.0	32.6 ± 6.2*	0.66	223.7 ± 89.9	17.6 ± 6.8*	0.99	219.1 ± 94.7	13.2 ± 7.7	0.83
**CCR**	193.5 ± 66.8	264.7 ± 77.3	32.5 ± 5.1*		229.7 ± 72.5	17.7 ± 4.8*		225.6 ± 75.4	17.6 ± 5.3*	
Vitamin B_{12} [µg/d]										
**ICR**	4.3 ± 2.4	4.1 ± 1.6	1.7 ± 9.2	0.89	3.4 ± 2.1	-24.8 ± 8.8*	0.96	3.7 ± 1.9	-9.5 ± 9.3	0.43
**CCR**	3.9 ± 1.8	3.8 ± 2.2	-8.8 ± 11.6		3.1 ± 1.3	-21.6 ± 8.6*		4.0 ± 1.9	-1.8 ± 11.0	
Calcium [mg/d]										
**ICR**	766.4 ± 05.1	799.3 ± 290.4	5.8 ± 5.4	0.90	683.1 ± 297.4	-11.5 ± 5.1*	0.79	686.0 ± 351.4	-13.6 ± 5.6*	0.06
**CCR**	689.8 ± 237.6	736.2 ± 206.3	5.1 ± 5.4		637.0 ± 216.7	-10.8 ± 5.4		708.6 ± 215.3	3.5 ± 5.4	
Iodide [µg/d]										
**ICR**	90.5 ± 44.8	80.3 ± 34.2	-10.3 ± 5.2	0.70	73.8 ± 47.2	-24.6 ± 6.4*	0.93	71.3 ± 28.0	-21.4 ± 7.1*	0.77
**CCR**	95.0 ± 51.3	80.8 ± 32.8	-11.8 ± 9.3		80.3 ± 37.7	-16.9 ± 8.0*		81.2 ± 39.4	-13.7 ± 10.2	
Iron [mg/d]										
**ICR**	9.1 ± 3.2	9.7 ± 2.9	10.6 ± 6.7	0.66	8.8 ± 3.2	-1.6 ± 6.0	0.98	9.2 ± 3.2	5.4 ± 5.4	0.84
**CCR**	9.3 ± 2.3	10.3 ± 2.7	9.2 ± 4.1*		9.0 ± 2.1	-2.3 ± 3.6		9.6 ± 3.1	0.2 ± 5.1	
Magnesium [mg/d]										
**ICR**	277.1 ± 92.0	322.7 ± 91.2	17.6 ± 4.4*	0.40	267.7 ± 93.8	-3.0 ± 4.6	0.75	282.0 ± 111.0	2.4 ± 4.7	0.57
**CCR**	276.1 ± 70.4	340.2 ± 87.6	19.3 ± 3.8*		274.4 ± 65.7	-0.2 ± 3.1		298.9 ± 87.6	6.8 ± 5.0	
Phosphorus [mg/d]										
**ICR**	1106.5 ± 416.2	1126.4 ± 362.2	4.7 ± 5.0	0.44	962.3 ± 410.5	-13.9 ± 5.0*	0.47	1016.5 ± 466.2	-6.5 ± 4.9	0.36
**CCR**	1025.1 ± 285.5	1112.2 ± 272.0	7.6 ± 4.5		948.5 ± 249.9	-8.4 ± 3.8*		1036.2 ± 258.2	0.5 ± 4.8	
Potassium [mg/d]										
**ICR**	2362.7 ± 846.1	2820.0 ± 827.6	20.5 ± 5.2*	0.99	2514.6 ± 884.5	8.2 ± 4.9	0.56	2470.3 ± 977.6	7.2 ± 5.1	0.55
**CCR**	2447.3 ± 601.3	2928.4 ± 741.0	17.4 ± 4.2*		2542.7 ± 632.1	3.9 ± 3.6		2702.3 ± 885.3	8.9 ± 4.5	
Zinc [mg/d]										
**ICR**	8.5 ± 3.2	8.7 ± 2.8	7.7 ± 6.0	0.74	7.4 ± 3.2	-12.3 ± 5.9*	0.92	8.0 ± 3.3	-0.8 ± 5.2	0.96
**CCR**	8.3 ± 2.3	8.9 ± 2.3	5.4 ± 4.7		7.5 ± 1.8	-10.1 ± 4.2*		8.3 ± 2.3	-3.0 ± 5.9	

	wk0	wk2	Change [%][1] wk0-wk2	P-value[2]	wk12	Change [%][1] wk0-wk12	P-value[2]	wk50	Change [%][1] wk0-wk50	P-value[2]

[1]Values are means ± SDs unless otherwise indicated. Changes are compared to baseline values and are shown as means of $\log_e$ percentage changes ± standard error of log percentage changes (%).
*Significant within group change over time compared to baseline at p <0.05 from paired t-test. Data were included from 98 participants (wk0 n = 49 for ICR and CCR; wk2 n = 45 for ICR, n = 46 for CCR; wk12 n = 47 for ICR, n = 46 for CCR; wk50 n = 41 for ICR, n = 38 for CCR). CCR, continuous calorie restriction; ICR, intermittent calorie restriction.
[2]P-values for time-treatment interactions were calculated with linear mixed models adjusted for age and sex.

Table S12. Macronutrient composition on non-restricted vs. restricted days at wk2, wk12 and wk50. [1]

		wk2	wk12	wk50
Protein [E%]	**NR day**	19.1 ± 3.0	17.6 ± 3.5	16.4 ± 2.7
	**R day**	23.7 ± 7.2	25.1 ± 7.5	24.7 ± 8.3
	P-value[2]	<.001	<.001	0.020
Protein [g/d]	**NR day**	79.9 ± 27.5	70.7 ± 27.6	69.1 ± 28.1
	**R day**	39.5 ± 13.8	41.9 ± 18.1	35.1 ± 17.9
	P-value[2]	<.001	<.001	0.005
Fat [E%]	**NR day**	31.2 ± 6.0	32.9 ± 6.1	34.9 ± 6.0
	**R day**	24.9 ± 10.4	23.8 ± 11.1	23.3 ± 13.2
	P-value[2]	<.001	<.001	0.014
Fat [g/d]	**NR day**	60.1 ± 24.3	61.0 ± 25.6	67.1 ± 27.3
	**R day**	20.5 ± 13.4	19.3 ± 13.5	13.7 ± 7.4
	P-value[2]	<.001	<.001	<.001
SFA [E%]	**NR day**	12.7 ± 2.9	12.6 ± 3.3	13.4 ± 3.3
	**R day**	9.9 ± 4.7	7.7 ± 3.8	8.2 ± 5.0
	P-value[2]	<.001	<.001	0.009
MUFA [E%]	**NR day**	10.3 ± 2.6	9.9 ± 2.8	10.1 ± 2.7
	**R day**	7.6 ± 3.7	6.9 ± 5.0	7.0 ± 4.8
	P-value[2]	<.001	<.001	0.07
PUFA [E%]	**NR day**	4.7 ± 1.5	4.8 ± 1.7	4.9 ± 2.1
	**R day**	4.4 ± 2.3	4.3 ± 2.7	5.2 ± 4.7
	P-value[2]	0.09	0.012	0.75
Carbohydrates [E%]	**NR day**	46.9 ± 5.9	47.1 ± 6.9	46.3 ± 6.8
	**R day**	47.1 ± 10.0	46.9 ± 9.9	47.6 ± 7.4
	P-value[2]	0.77	0.40	0.82
Carbohydrates [g/d]	**NR day**	200.6 ± 78.7	187.4 ± 62.8	195.6 ± 76.6
	**R day**	80.0 ± 29.2	77.8 ± 29.0	65.4 ± 22.6
	P-value[2]	<.001	<.001	<.001
Sugar [g/d]	**NR day**	77.4 ± 39.7	72.2 ± 28.9	72.3 ± 32.5
	**R day**	41.7 ± 17.3	41.5 ± 20.7	30.9 ± 10.6
	P-value[2]	<.001	<.001	<.001
Fiber [E%]	**NR day**	2.8 ± 0.8	2.4 ± 0.8	2.3 ± 0.8
	**R day**	4.3 ± 1.3	4.2 ± 1.7	4.5 ± 1.9
	P-value[2]	<.001	<.001	<.001
Fiber [g/d]	**NR day**	24.8 ± 9.8	20.5 ± 9.5	20.2 ± 9.3
	**R day**	14.7 ± 4.5	14.8 ± 6.8	12.7 ± 5.8
	P-value[2]	<.001	<.001	0.047

[1]Values are means ± SDs unless otherwise indicated. Data were included from 49 participants (wk2 n = 45 for NR and R days; wk12 n = 47 for NR days, n = 44 for R days; wk50 n = 41 for NR days, n = 12 for R days). CCR, continuous calorie restriction; ICR, intermittent calorie restriction; NR, non-restricted; R, restricted.

[2]P-values for differences between NR and R days at each time point were calculated by paired t-test.

Table S13. Micronutrient intake on non-restricted vs. restricted days at wk2, wk12 and wk50. [1]

		wk2	wk12	wk50
Vitamin A (Retinol) [mg/d]	**NR day**	0.7 ± 2.0	0.6 ± 1.9	0.4 ± 0.5
	**R day**	0.2 ± 0.1	0.1 ± 0.1	0.1 ± 0.1
	P-value[2]	<.001	<.001	0.004
β-carotene [mg/d]	**NR day**	7.4 ± 5.7	5.6 ± 5.3	4.1 ± 3.8
	**R day**	10.1 ± 7.6	7.9 ± 7.2	7.6 ± 6.1
	P-value[2]	<.001	0.041	0.018
α-tocopherol [mg/d]	**NR day**	9.4 ± 3.8	8.2 ± 3.8	7.9 ± 2.9
	**R day**	5.7 ± 2.6	5.7 ± 3.4	5.7 ± 2.7
	P-value[2]	<.001	<.001	0.20
Vitamin C [mg/d]	**NR day**	142.1 ± 89.2	125.1 ± 68.7	121.3 ± 77.6
	**R day**	134.0 ± 65.4	130.0 ± 78.3	155.3 ± 95.5
	P-value[2]	0.60	0.45	0.13
Vitamin D [µg/d]	**NR day**	2.6 ± 2.9	1.9 ± 1.9	1.9 ± 1.9
	**R day**	2.6 ± 3.3	1.0 ± 1.8	1.7 ± 3.4
	P-value[2]	0.05	<.001	0.14
Thiamin [mg/d]	**NR day**	1.2 ± 0.4	1.0 ± 0.4	1.0 ± 0.5
	**R day**	0.7 ± 0.3	0.6 ± 0.3	0.5 ± 0.2
	P-value[2]	<.001	<.001	0.002
Riboflavin [mg/d]	**NR day**	1.4 ± 0.5	1.2 ± 0.5	1.1 ± 0.5
	**R day**	1.0 ± 0.3	0.9 ± 0.4	0.7 ± 0.4
	P-value[2]	<.001	<.001	0.008
Niacin [mg/d]	**NR day**	33.1 ± 11.1	27.2 ± 11.2	26.1 ± 11.7
	**R day**	18.3 ± 6.7	16.6 ± 7.8	14.2 ± 7.4
	P-value[2]	<.001	<.001	0.010
Vitamin B_6 [mg/d]	**NR day**	1.7 ± 0.7	1.4 ± 0.5	1.3 ± 0.6
	**R day**	1.0 ± 0.3	1.0 ± 0.4	0.9 ± 0.4
	P-value[2]	<.001	<.001	0.12
Folate [µg/d]	**NR day**	266.5 ± 92.7	225.1 ± 93.9	217.7 ± 95.4
	**R day**	224.6 ± 75.9	221.3 ± 117.1	204.8 ± 100.4
	P-value[2]	0.010	0.31	0.69
Vitamin B_{12} [µg/d]	**NR day**	4.8 ± 2.2	3.9 ± 2.5	3.8 ± 2.0
	**R day**	2.4 ± 1.6	2.1 ± 1.3	1.7 ± 1.3
	P-value[2]	<.001	<.001	0.06
Calcium [mg/d]	**NR day**	887.2 ± 357.9	726.8 ± 312.9	701.6 ± 355.8
	**R day**	583.2 ± 225.3	544.6 ± 296.1	398.5 ± 149.8
	P-value[2]	<.001	<.001	0.003
Iodide [µg/d]	**NR day**	89.3 ± 45.1	79.6 ± 49.5	71.5 ± 28.7
	**R day**	57.9 ± 28.5	57.9 ± 55.4	63.8 ± 78.3
	P-value[2]	<.001	<.001	0.18
Iron [mg/d]	**NR day**	11.2 ± 3.9	9.8 ± 3.9	9.5 ± 3.4
	**R day**	6.2 ± 1.9	5.9 ± 2.2	5.0 ± 2.5
	P-value[2]	<.001	<.001	0.003
Magnesium [mg/d]	**NR day**	359.9 ± 111.5	292.1 ± 108.0	288.4 ± 114.6
	**R day**	231.9 ± 73.1	201.3 ± 73.8	179.1 ± 88.8
	P-value[2]	<.001	<.001	0.002
Phosphorus [mg/d]	**NR day**	1309.3 ± 484.6	1072.8 ± 461.9	1048.6 ± 487.4
	**R day**	680.3 ± 220.8	652.5 ± 296.5	515.5 ± 204.3

		wk2	wk12	wk50
	P-value[2]	<.001	<.001	<.001
Potassium [mg/d]	**NR day**	3060.3 ± 1026.4	2651.3 ± 996.6	2506.9 ± 1018.1
	**R day**	2236.5 ± 667.1	2175.8 ± 897.2	1780.4 ± 851.5
	P-value[2]	<.001	<.001	0.06
Zinc [mg/d]	**NR day**	10.2 ± 3.6	8.4 ± 3.8	8.3 ± 3.3
	**R day**	4.9 ± 1.5	4.9 ± 2.1	3.2 ± 1.4
	P-value[2]	<.001	<.001	<.001

[1]Values are means ± SDs unless otherwise indicated. Data were included from 49 participants (wk2 n = 45 for NR and R days; wk12 n = 47 for NR days, n = 44 for R days; wk50 n = 41 for NR days, n = 12 for R days). CCR, continuous calorie restriction; ICR, intermittent calorie restriction; NR, non-restricted; R, restricted.
[2]P-values for differences between NR and R days at each time point were calculated by paired t-test.

Table S14. Intake of foods [g/d] on non-restricted vs. restricted days at wk2, wk12 and wk50. [1]

		wk2	wk12	wk50
Vegetables and vegetable products [g/d]	**NR day**	265.2 ± 152.0	251.6 ± 218.7	179.9 ± 123.9
	**R day**	329.7 ± 141.3	382.4 ± 261.5	335.2 ± 288.0
	P-value[2]	0.002	0.002	0.004
Fruits and fruit products [g/d]	**NR day**	188.7 ± 112.5	182.6 ± 117.9	178.3 ± 151.7
	**R day**	130.3 ± 77.6	131.1 ± 86.4	80.3 ± 76.1
	P-value[2]	<.001	0.035	0.81
Bread [g/d]	**NR day**	133.6 ± 84.7	123.9 ± 68.4	122.7 ± 83.1
	**R day**	25.5 ± 25.4	28.9 ± 35.7	34.7 ± 32.0
	P-value[2]	<.001	<.001	0.016
Grains and grain products, rice [g/d]	**NR day**	37.7 ± 34.8	24.5 ± 22.8	25.7 ± 26.0
	**R day**	33.0 ± 44.5	18.9 ± 34.4	8.8 ± 16.8
	P-value[2]	0.08	0.41	0.84
Potatoes and starchy foods, mushrooms [g/d]	**NR day**	41.8 ± 43.6	40.1 ± 46.0	41.6 ± 40.3
	**R day**	44.9 ± 64.3	31.5 ± 69.5	45.4 ± 67.1
	P-value[2]	0.26	0.06	0.96
Milk, dairy products and cheese [g/d]	**NR day**	203.8 ± 177.3	194.4 ± 141.5	177.2 ± 159.3
	**R day**	172.3 ± 118.2	215.7 ± 178.0	171.3 ± 104.1
	P-value[2]	0.41	0.38	0.89
Beef, veal, pork, mutton [g/d]	**NR day**	20.7 ± 22.7	17.3 ± 31.6	15.0 ± 23.8
	**R day**	0.0 ± 0.0	8.2 ± 22.0	0.0 ± 0.0
	P-value[2]	.	0.17	.
Game, poultry, offal [g/d]	**NR day**	26.5 ± 28.2	23.4 ± 32.9	14.7 ± 21.3
	**R day**	9.9 ± 22.3	16.4 ± 35.9	21.6 ± 41.8
	P-value[2]	0.017	0.09	0.27
Sausage and processed meat [g/d]	**NR day**	32.8 ± 27.2	26.1 ± 27.4	41.1 ± 33.1
	**R day**	10.3 ± 22.7	14.6 ± 25.3	18.2 ± 43.7
	P-value[2]	0.82	0.34	0.61
Fish and seafood [g/d]	**NR day**	23.7 ± 34.6	15.9 ± 22.3	10.0 ± 16.7
	**R day**	19.0 ± 32.6	8.4 ± 22.9	20.2 ± 37.7
	P-value[2]	0.44	0.26	.
Eggs and egg products, pasta [g/d]	**NR day**	30.7 ± 34.5	35.4 ± 37.9	34.6 ± 38.7
	**R day**	27.6 ± 38.4	23.4 ± 31.3	25.0 ± 42.1
	P-value[2]	0.026	0.35	0.30
Fats and oils [g/d]	**NR day**	10.9 ± 8.2	11.6 ± 9.0	13.6 ± 12.1

		wk2	wk12	wk50
	**R day**	1.8 ± 3.1	3.0 ± 7.1	7.7 ± 17.4
	P-value[2]	0.003	0.84	0.99
Legumes, nuts and seeds [g/d]	**NR day**	12.8 ± 21.4	15.0 ± 21.6	7.0 ± 12.0
	**R day**	5.4 ± 16.9	5.0 ± 13.0	0.5 ± 1.5
	P-value[2]	0.37	0.019	0.64
Bakery products, cakes and pastry [g/d]	**NR day**	26.3 ± 33.3	31.7 ± 34.8	51.6 ± 44.8
	**R day**	0.0 ± 0.0	1.8 ± 8.5	0.0 ± 0.0
	P-value[2]	.	0.05	.
Sweets, sugar and ice cream [g/d]	**NR day**	25.3 ± 34.5	27.0 ± 27.2	27.5 ± 37.1
	**R day**	5.0 ± 13.1	2.2 ± 5.7	4.0 ± 9.0
	P-value[2]	0.042	<.001	0.96

[1]Values are means ± SDs unless otherwise indicated. Data were included from 49 participants (wk2 n = 45 for NR and R days; wk12 n = 47 for NR days, n = 44 for R days; wk50 n = 41 for NR days, n = 12 for R days). CCR, continuous calorie restriction; ICR, intermittent calorie restriction; NR, non-restricted; R, restricted.

[2]P-values for differences between NR and R days at each time point were calculated by paired t-test.

References

1. Schübel R, Nattenmuller J, Sookthai D, Nonnenmacher T, Graf ME, Riedl L, Schlett CL, von Stackelberg O, Johnson T, Nabers D, et al. Effects of intermittent and continuous calorie restriction on body weight and metabolism over 50 wk: a randomized controlled trial. Am J Clin Nutr 2018;108(5):933-45.
2. Penningten Biomedical Research Center, Lasater RS, Starling JK. Penningten Biomedical Research Center weight loss predictor. Internet: https://www.pbrc.edu/research-and-faculty/calculators/weight-loss-predictor/about/ (accessed 22 March 2021).
